# The effects of maternal voice on pain during placement of peripherally inserted central catheter in neonates

**DOI:** 10.3389/fpain.2024.1483317

**Published:** 2024-10-29

**Authors:** Audrey Flours, Fabienne Mons, Antoine Bedu, Thomas Lauvray, Anne-Laure Blanquart, Jean-Baptiste Woillard, Audrey Mowendabeka, Vincent Guigonis, Laure Ponthier

**Affiliations:** ^1^ Department of Neonatal Intensive Care Unit, CHU, Limoges, France; ^2^ Pharmacology and Toxicology, Univ. Limoges, INSERM, Limoges, France; ^3^ Department of Pharmacology and Toxicology, CHU, Limoges, France

**Keywords:** neonates, pain, maternal, voice, central catheter

## Abstract

**Background:**

Peripherally inserted central catheter (PICC) are a necessary procedure for preterm newborns care. Despite the use of analgesic treatments, its insertion can be painful. Our objective was to study the effect of maternal voice on pain during PICC insertion.

**Method:**

We conducted a pre post study for 2 years. Pain was compared between the two groups (with/without maternal presence) using a neonatal pain scale (FANS). Infection rate, procedure time, number of failures, mothers’ anxiety and caregivers’anxiety were compared between the two groups.

**Results:**

Ninety neonates were eligible. Finally, 63 neonates were included. Thirty-four placements were realized without maternal voice (first period) and 29 with maternal voice (second period). Mean FANS during PICC placement was lower in the maternal voice group than in the control group (1.15 ± 1.27 vs. 1.41 ± 1.49, *p* = 0.033). The FANS was also lower in the maternal voice group during the time of the first cutaneous effraction (*p* = 0.032). There was no significant difference between the two groups concerning the other outcomes.

**Conclusion:**

Maternal voice added to conventional care decreased acute pain during PICC insertion without increasing infection rate, number of failures or procedure time.

## Introduction

Worldwide, prematurity concerns about 15 millions newborns each year (1). Complications in preterm newborns are frequent and require specialized care. Optimal growth limits short-term and long-term complications such as retinopathy, chronic lung disease and poor long-term neurodevelopmental outcomes (2–4). Parenteral nutrition is essential to obtain optimal growth in low-birth-weight infants. Parenteral nutrition is administrated via central catheters, such as peripherally inserted central catheters (PICC). However, this procedure is one of the five most painful procedures in neonatology and for which the number of attempts is the highest, therefore participating to frequent painful stimulation (5). Multiple painful and stressful events have multiple consequences: more sensitivity to future pain reaction (6), heart frequency increase, decrease oxygen rate saturation (7), higher stress and neurodevelopment disorders (8). Pharmacological and non-pharmacological interventions such as swaddling, nonnutritive sucking, skin-to-skin care, breastfeeding, sucrose or glucose can be used in association to increase the efficacy of analgesia during painful procedures, and the use of pain scales is also important to assess the effectiveness of these interventions (9–13). As far as PICC placement is concerned, the best lowering pain strategy is yet to be determined.

Maternal voice is an additional non-pharmacological intervention to limit child's pain and has many benefit impacts. Indeed, the auditory system is functional during the first trimester and the fetus can perceive sound and react to it (14, 15). Functional magnetic resonance imaging and high-density electroencephalography have shown that preterm infants and full neonates could differentiate maternal and stranger voices (16). Maternal voice speaking or singing have positive impacts on preterm infants’ physiologic responses (increase oxygen saturation, decrease heart rate and respiratory rate), but also improves neurobehavioral outcomes, improves feeding tolerance or full enteral feeding step, improves quiet behavioral states and the maturation of their autonomic nervous system (17–19). Studies have reported that maternal voice reduces neonatal pain during heel stick or venipuncture procedure (20–23), therefore such a strategy could be tested during PICC insertion. On the other hand, maternal presence during PICC insertion may lead to adverse effects such as anxiety in caregivers or mothers and higher failure rate. No study has evaluated the impact of maternal voice on newborn's pain during PICC placement.

The objective of this study was (i) to evaluate the impact of maternal voice on the level of pain of their child during PICC placement, (ii) to evaluate the side effects of maternal presence during this procedure, (iii) to evaluate mothers and caregivers’ anxiety during this procedure.

## Material and methods

This prospective observational study was conducted on neonates hospitalized in the Neonatal intensive care unit of Limoges university hospital (France) from September 2020 to August 2022. This pre post study compared two periods: a first period when the mothers were not allowed to be present during PICC insertion (September 2020 to August 2021) (=control group) and a second one when they were authorized and participated with her voice (see details below) (September 2021 to August 2022) (=maternal voice group). This corresponds to a change of practice in our ward after a medical and nurse staff decision.

Neonates born before 35 amenorrhea weeks who required a PICC insertion were included. Neonates were excluded if they have a pathology that could interfere with the measured pain score: congenital heart disease, grade 3 or 4 intraventricular hemorrhage, hemodynamic instability, necrotizing enterocolitis, sedation use during the procedure or 24 h before. In the second period, an additional exclusion criterion was the mother's inability or unwillingness to be present, or if she wished to be present but did not wish to speak or sing during the procedure.

The primary outcome was to compare the pain between the control group and the maternal voice group. The judgment criterion for primary outcome was the mean of all recorded FANS (Faceless acute Neonatal pain scores) (24) during the procedure. FANS was recorded at specific time points during the procedure: during the caregivers’ hand washing, during skin disinfection of the site, at each cutaneous effraction, each time the needle is removed, during catheter ascension, during the radiographic catheter placement control, during bandage.

Secondary outcomes were to compare: pain at several time points of the procedure, duration of PICC placement, the number of cutaneous effraction for PICC placement, failure rate, mothers’ or the caregiver’ anxiety and central-line-associated bloodstream infections (CLASBI). The duration of PICC insertion was defined by the time elapsed between the start of caregiver hand washing and the end of catheter dressing. The failure rate was defined as the absence of successful catheter placement after three cutaneous effractions. The mothers’ anxiety and the caregivers’ anxiety were assessed at the end of each procedure using a numeric scale rated from 0 to 10. The central-line-associated bloodstream infections (CLASBI) was defined as a laboratory-confirmed bloodstream infection, not related to an infection from another site, that develops from 48 h after the placement of a central line to 48 h after its removal (25).

A pain management protocol was administered to every patient, according to local practice: swaddling the child with the use of non-nutritive suction including oral administration of sucrose and paracetamol. To limit the pain and the risk of CLASBI, only three cutaneous effractions were allowed during each procedure. According to the French guidelines (26), PICC placements were performed with a physician and a nurse under aseptic precautions. A third person intervened for administration of analgesic measures and scored the pain score. A maximum of four people were allowed in the room during the procedure to limit the risk of CLASBI.

We chose the FANS to evaluate pain during this procedure without taking into account the facial expression which is not accessible during the PICC placement (the child's face is covered by a sterile drape) (24). The pain score was scored by one of the 15 childcare assistants, who had theoretical and practical training to score/record it. During the procedure, to better record the score, the childcare assistant positioned her hands on the child under the sterile drapes to perceive the movements of the limbs. Heart rate and oxygen saturation were measured using a monitor allowing recordings.

During the second period when mothers were authorized, they could be present on a voluntary basis. They choose to read tales, or tell her own stories, or sing or talk to her child. The mothers were asked to simply wash their hands after putting an overcoat and a protection on their hair. They were placed in a comfortable chair and were systematically reassured by the present nursing staff.

This study received the approval of the local ethics committee (n° 495-2021-151). Informed consent was obtained from the parents of all newborns included in this study.

### Statistical analyses

Statistical analyses were performed using R software version 4.1.1. Descriptive statistics were given as means and standard deviations for quantitative variables. Categorical variables were presented as percentages. Mann–Whitney test was used to compare continuous variable. Qualitative variables were compared using Chi2 or Fisher's exact test. Values of *p* < 0.05 were considered statistically significant.

### The non-normality of FANS was confirmed using a Shapiro–Wilk test

A Linear Mixed effect (LME) model was tested to investigate the influence of the maternal voice during repeated cutaneous effractions on log transformed FANS. Different structural models were tested (random intercept or slope with and without intermodel correlation), and the best model was selected based on the Akaike information criterion (AIC). We tested in univariate analysis the following variables: maternal voice, number of cutaneous effraction. Covariates associated with a *P* value < 0.2 in the univariate analysis were considered clinically relevant and plausible and were, therefore, included in the multivariate intermediate model. The final model was selected using a backward stepwise process based on the AIC. Finally, in the final model, linearity, homoscedasticity, normality of residuals, absence of influential data points, and independence were checked.

## Results

**Figure 1:**
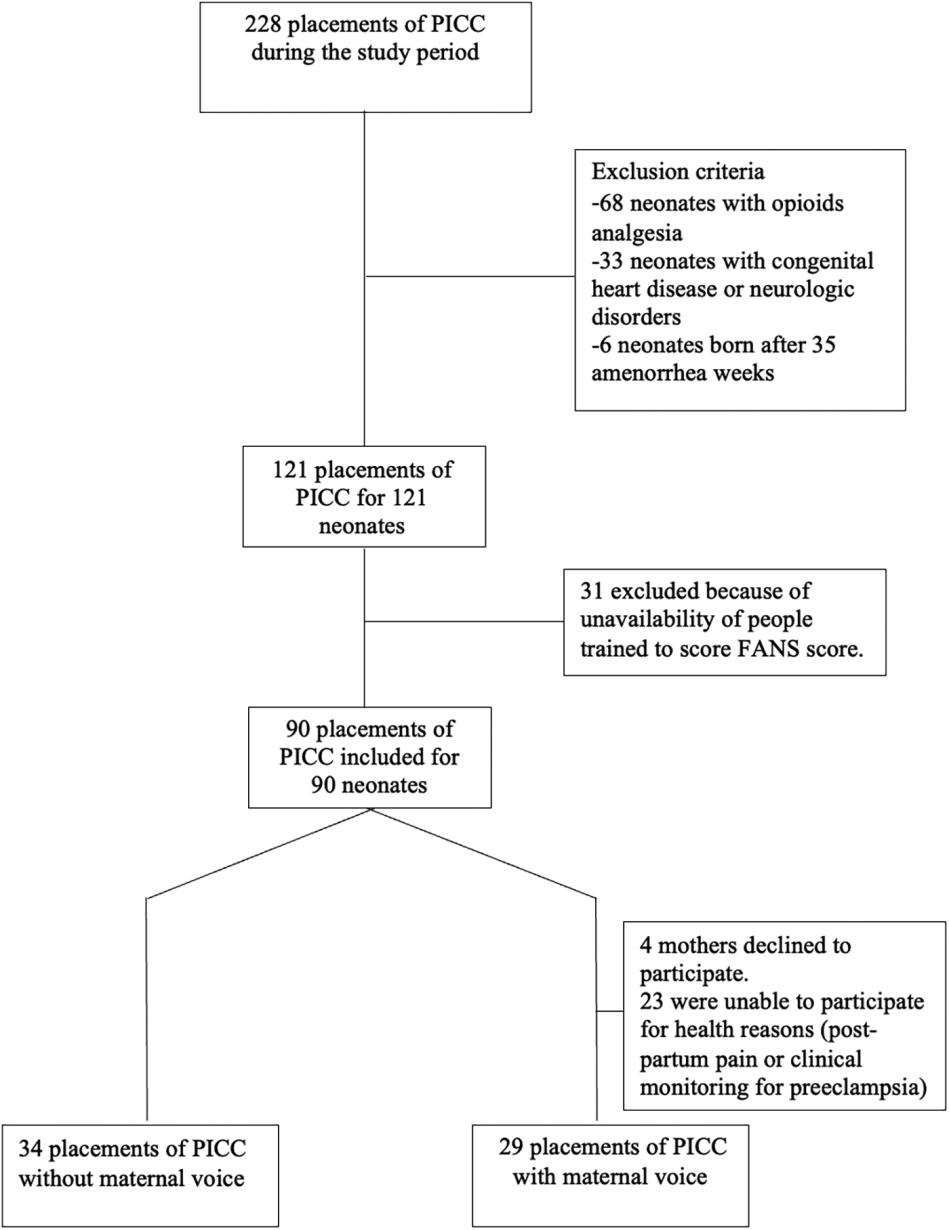
Flow chart of the study.

Out of ninety eligible neonates, a total of sixty-three PICC placements were included during the study period (Figure 1). Thirty-four placements were realized in the control group and 29 in the maternal voice group. During the second period, 23 mothers were unable to participate for health reasons (post-partum pain or clinical monitoring for preeclampsia) and 4 mothers declined to participate. The clinical characteristics were compared between the 2 groups and no statistical difference was present (Table 1).

**Table 1:** Clinical characteristics of the patients.

	Total *N* = 63	Control group *N* = 34	Maternal voice group *N* = 29	*p*
Sex male	35 (41.5%)	19 (44.1%)	16 (55.2%)	1.0
Birth term (amneorrhea weeks)	29.1 ± 2.55	29.4 ± 2.81	28.7 ± 2.8	0.34
Birth weight (grams)	1,165 ± 365	1,196 ± 355	1,129 ± 380	0.47
Days of PICC placement	3.75 ± 5.6	4.88 ± 6.7	2.41 ± 3.6	0.069
Percentile weight (percentile)	46.2 ± 29.1	46.6 ± 27.7	45.9 ± 31.1	0.93
Height (centimeter)	36.7 ± 5.93	37.3 ± 7.4	35.9 ± 3.6	0.36
Percentile height (percentile)	32.8 ± 23.6	30.2 ± 20.8	35.9 ± 26.5	0.36
Intrauterine growth retardation	14 (22.2%)	6 (17.6%)	8 (27.6%)	0.521
Maternal corticosteroid therapy	54 (85.7%)	32 (94.1%)	27 (93.1%)	0.87
APGAR at 5 min	8.9 ± 1.7	8.97 ± 1.6	8.75 ± 1.8	0.62
Mode of delivery				0.253
Vaginal delivery	20 (31.7%)	8 (23.5%)	12 (41.4%)	
Caesarean section	43 (68.3%)	26 (76.5%)	17 (58.6%)	
Antibiotic therapy during the first days of life	37 (58.7%)	17 (50.0%)	20 (69%)	0.21
Surfactant:	24 (38.1%)	12 (35.3%)	12 (41.4%)	0.81
Maternal smoking	12 (19.0%)	5 (14.7%)	7 (24.1%)	0.44
Maternal intake of toxic substances	1 (1.6%)	0 (0%)	1 (3.5%)	0.39
Neonatal Ventilatory support during the placement				0.57
Oxygen	2 (3.2%)	2 (5.9%)	1 (3.5%)	
Non invasive ventilation	54 (85.8%)	27 (79.4%)	27 (93.1%)	

Numeric Data are presented as a mean ± standard deviation (unless otherwise indicated).

Differences between continuous variables were determined by the Mann–Whitney *U*.

Differences between categorical variables were determined by the *X*^2^ test.

The mean FANS during the PICC placement was lower in the maternal voice group than in the control group (1.15 ± 1.27 vs. 1.41 ± 1.49, *p* = 0.033). During this study, a total of 606 scores were recorded and the mean for the FANS was 1.29 ± 1.39 in our overall population.

When we looked at each step of the procedure there were no differences between the two groups except for the FANS recorded during the time of the first cutaneous effraction: the mean FANS was lower for the maternal voice group (*p* = 0.032). These data are summarized in Table 2.

**Table 2:** Mean of FANS in each group at each step of the procedure.

Step of the procedure		All	Control group	Maternal voice group	*p*
Hand wash		0.61 ± 0.94	0.65 ± 0.98	0.58 ± 0.90	0.786
Skin disinfection		1.58 ± 1.36	1.66 ± 1.45	1.50 ± 1.26	0.657
First attempt *N* = 82 neonates	Cutaneous effraction	1.40 ± 1.53	1.76 ± 1.74	0.97 ± 1.12	0.032*
	Catheter ascension	1.26 ± 1.43	1.54 ± 1.62	0.91 ± 1.06	0.107
	Needle removal	0.94 ± 1.13	1.06 ± 1.20	0.79 ± 1.05	0.353
Second Attempt *N* = 58 neonates	Cutaneous effraction	1.53 ± 1.45	1.70 ± 1.61	1.35 ± 1.27	0.436
	Catheter ascension	1.74 ± 2.10	1.90 ± 2.25	1.45 ± 1.86	0.560
	Needle removal	1.02 ± 1.08	1.17 ± 1.23	0.85 ± 0.88	0.322
Third attempt *N* = 45 neonates	Cutaneous effraction	2.16 ± 1.46	1.85 ± 1.50	2.53 ± 1.37	0.159
	Catheter ascension	1.76 ± 1.64	1.60 ± 1.55	2.00 ± 1.83	0.576
	Needle removal	1.08 ± 1.19	1.25 ± 1.41	0.88 ± 0.86	0.338
X-ray	1.12 ± 1.30	1.28 ± 1.17	0.96 ± 1.20	0.347	
Bandage	1.27 ± 1.30	1.36 ± 1.29	1.17 ± 1.34	0.626	

Numeric Data are presented as a mean ± standard deviation (unless otherwise indicated).

Differences between continuous variables were determined by the Mann–Whitney *U*.

**Figure 2:**
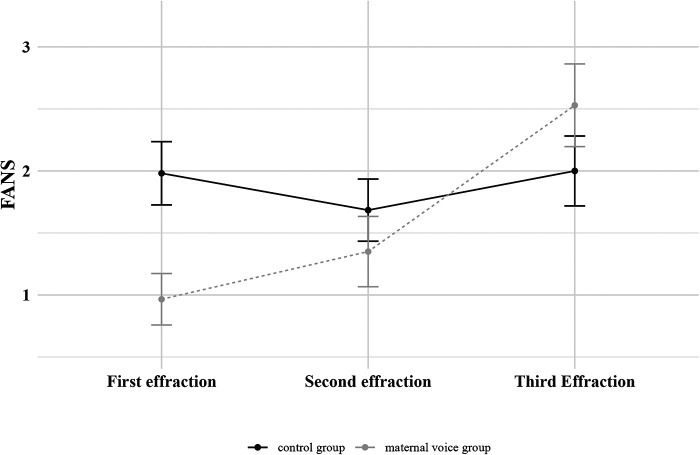
Evolution of the FANS between the 2 groups (control group or maternal voice group) according to the number of cutaneous effractions. Means with standard error of the mean are represented.

We studied the evolution of FANS between the 2 groups according to the number of cutaneous effractions (Figure 2). The beneficial effect of maternal voice on FANS seemed to decrease with each cutaneous effraction. This was confirmed by LME (Table 3).

**Table 3:** Multivariate generalized linear model for FANS.

	Mean	Standard error	*p*. value
Intercept	0.822	0.099	<0.001
Maternal voice for first cutaneous effraction	−0.298	0.145	0.040
Second cutaneous effraction with maternal voice	0.132	0.169	0.44
Second cutaneous effraction without maternal voice	0.028	0.115	0.8
Third cutaneous effraction with maternal voice	0.450	0.179	0.014
Third cutaneous effraction without maternal voice	0.159	0.121	0.19

The marginal effects were calculated at the means of the independent variables.

The mean PICC placement duration was 72.3 ± 25.5 min in the control group vs. 71.2 ± 20.4 min in the maternal voice group (*p* = 0.851). The numbers of cutaneous effraction for PICC placement were 1.4 ± 0.7 for the control group vs. 1.2 ± 0.5 for the maternal voice group (*p* = 0.098). The failure rate was 17.6% (six PICC placements) in the control group vs. 17.9% (five PICC placements) in the maternal voice group (*p* = 1.0). Concerning the mothers’ anxiety, there was no significant difference in scores between the two groups: 5.3 ± 2.3 in the control group vs. 4.2 ± 2.4 in the maternal voice group (*p* = 0.135). Concerning the nurses’ anxiety, there was a significant difference in scores between the two groups, with a lower score in the maternal voice group than in the control group (1.96 ± 2.26 vs. 3.68 ± 1.96, *p* = 0.007). Concerning the doctors’ anxiety, there was not significant difference in scores between the two groups: 2.93 ± 1.97 in the control group vs. 1.97 ± 1.77 in the maternal voice group (*p* = 0.062). Concerning the CLASBI, there were six infections (21.4%) in the control group vs. two infections (8.33%) in the maternal voice group (*p* = 0.260).

## Discussion

To our knowledge, this is the first study demonstrating that maternal voice reduces pain in newborns during PICC insertion. This effectiveness is especially pronounced during the initial cutaneous effraction. No side effect to maternal voice was found, particularly no increase in maternal or caregivers’ anxiety, and no increase in failure rate.

Maternal voice has already demonstrated its effectiveness in similar procedures such as during heel stick or venipuncture procedure (20–23). Our results are of interest as PICC insertion duration is longer than venipuncture and parental presence during this procedure is rare (27). Nevertheless, even if pain scores were rather low throughout the procedure, we have shown that effectiveness decreased with each venipuncture. Therefore it remains important to combine multiple pharmacological and non-pharmacological interventions to limit pain (9, 10, 13) as well as train caregivers to limit repeated cutaneous effraction (28). Overall, there is a benefit to the mother's voice during this invasive procedure. Moreover, involving parents in neonatal care benefits the neonates but also the parents themselves, increasing bonding and attachment as they are more present with their child (29).

We were concerned about increasing the anxiety of the mothers by implicating them in such a long procedure which have a risk of failure. Indeed, hospitalization of their neonates induces stress and anxiety in the mothers, potentially leading to a state of anxiety-depression in the mother and adversely impacting the neuromotor development of their children (30). We found no differences in mother's anxiety scores between the two groups. The lower nurse anxiety scores in the maternal voice group might be explained by the serene atmosphere produced by the mother's reassuring voice.

We chose to use the FANS in order not to take account of facial expression, which is impossible to score in this clinical situation (24). The FANS had some advantages as it allowed for consideration of vocal reactions’ reduction or cardiorespiratory instability which may be encountered in procedural pain. One of the limits of this scale is that the FANS is not the most used scale in neonatology and requires trained caregivers. Nevertheless, once caregivers are trained, this scale demonstrates real value (24).

Our study had some limits. Firstly, as a pre post study, it was not randomized. Indeed, we already had allowed parental presence in our unit during intubation or cardio-pulmonary resuscitation. As such, forbidding maternal presence at random during PICC insertion was considered ethically questionable for the staff and could not be enforced by caregivers.

Secondly, the pain difference between groups was not very high and one can question its clinical relevance. However, since maternal voice has no side effects, it would be regrettable not to involve mothers. Of note, four mothers declined to participate. A limit to mother's participation was persistent health problems that limit their presence in the early days after birth. It is important to train healthcare providers to support parents and encourage them in their role.

Thirdly, pain assessment was based solely on a clinical score. To support this clinical impact, it would have been interesting to have a multimodal assessment of pain with parasympathetic evaluation or electroencephalography (31, 32).

Fourthly, in the maternal voice group, PICC placements were performed more rapidly after birth. This may have reduced the CLASBI rate in the maternal group, leading to an underestimation of the infectious risk associated with the maternal presence. But, as a Cochrane review did not conclude that early removal of umbilical venous catheters reduced the risk of infection (33), the absence of difference in CLASBI rate between the two groups leads us to continue to favor the presence of mothers during PICC insertion.

Finally, the scale used for anxiety is questionable as it lacks precision. It would have been interesting to assess this anxiety more accurately with a specific score or a psychological evaluation, for example.

In conclusion, our study showed that maternal voice decreased acute pain during PICC placement, particularly during the first cutaneous effraction without adverse effects, notably without raising caregivers’ or mothers’ anxiety. Even though studies examining this strategy with a multimodal assessment of pain could be proposed, we believe that these results justify involving mothers during PICC procedures. However, the predominant effect of maternal voice on the first cutaneous effractions still requires a multifaceted approach to pain and trained teams to minimize the number of punctures.

## Data Availability

The raw data supporting the conclusions of this article will be made available by the authors, without undue reservation.
